# Effectiveness of a heart disease blended learning program in physiotherapy students: a prospective study

**DOI:** 10.3389/fcvm.2023.1303997

**Published:** 2023-11-22

**Authors:** Elena Marques-Sule, David Hernández-Guillén, Natalia Cezón-Serrano, Laura Arjona-Tinaut, Amalia Sillero-Sillero, Juan Luis Sánchez González, Ana Cobos-Rincón, Raúl Juárez-Vela, Elena Muñoz-Gómez

**Affiliations:** ^1^ Physiotherapy in Motion, Multispecialty Research Group (PTinMOTION), Department of Physiotherapy, University of Valencia, Valencia, Spain; ^2^ Department of Physiotherapy, Faculty of Physiotherapy, University of Valencia, Valencia, Spain; ^3^ Group of Physiotherapy in the Ageing Process: Social and Health Care Strategies, Department of Physiotherapy, University of Valencia, Valencia, Spain; ^4^ Escola Superior D'Infermeria del Mar (ESIMar), Parc de Salut Mar, Universitat Pompeu Fabra, Barcelona, Spain; ^5^ Social Determinants and Health Education Research Group, IMIM (Hospital del Mar Medical Research Institute), Barcelona, Spain; ^6^ Department of Nursing and Physiotherapy, Instituto de Investigación Biomédica de Salamanca (IBSAL), University of Salamanca, Salamanca, Spain; ^7^ Department of Nursing, Faculty of Health Sciences, University of La Rioja, Research Group GRUPAC, Logroño, Spain

**Keywords:** physiotherapy students, cardiology, blended learning, education, teaching methods

## Abstract

**Background:**

In recent years, novel teaching methodologies have been emerging with the aim of improving student learning. One of them is known as Blended Learning. BL allows educators to integrate elements of traditional face-to-face teaching with tailored online learning modalities, integrating the distinct strengths of both methodologies.

**Purpose:**

To determine the effectiveness of a heart disease blended learning program in undergraduate physiotherapy students.

**Methods:**

124 participants (average age 21.20 ± 4.67 years, 58.87% female) performed an 8-week heart disease blended learning program that included face-to-face classes and online resources. Knowledge was assessed at baseline, at 4, 8, 12 and 20 weeks. Motivation and engagement were assessed at 4 and 8 weeks. Design of the instructions and learning behaviors were measured at 8 weeks. Finally, 108 subjects completed the study

**Results:**

Knowledge significantly increased mid-program (*p *= 0.02), at the end of the program (*p* < 0.001), at 12 weeks (*p* < 0.001) and 20 weeks (*p *= 0.001). After the intervention, a high intrinsic motivation was shown (5.60 ± 0.80)) over 7), whilst extrinsic motivation scored 4.24 ± 0.97 over 7.Finally, engagement (3.98 ± 0.52) over 5), design of the instructions (4.15 ± 0.62) over 5) and learning behaviors (70.51 ± 36.08) downloads, 28.97 ± 16.09) topics visited, and online questionnaires scored 7.67 ± 1.60) over 10) reported adequate scores.

**Conclusion:**

This program seems to be an appropriate methodology in future physiotherapists, since it improved knowledge and participants exhibited a high motivation and an adequate engagement, design of the program instructions and learning behaviors.

## Introduction

The rapid technological evolution experienced recently has meant significant progress, resulting in greater accessibility to knowledge generating changes in the needs of society (1). In the same way, these changes must also be implemented in higher education to promote active learning and thus improve the theoretical and practical results of future health professionals (2). Accordingly, lately, a new type of educational approach known as blended learning (BL) has emerged (1, 3). It has the potential to foster innovative and flexible learning opportunities (4). Moreover, BL further allows educators to integrate elements of traditional face-to-face teaching with tailored online learning modalities (4, 5),integrating the different strengths of both methodologies (6).

One of the principles that should pursue education is to attempt to improve knowledge acquisition. A recent meta-analysis by Li et al. (7) demonstrated the high impact of BL on knowledge compared to face-to-face interventions. Moreover, other studies observed improvements in knowledge (8) and integration of theory and practice (9–11) following BL interventions.

Motivating future healthcare professionals is necessary to improve the acquisition of knowledge. Previous studies have strongly suggested that BL promotes such motivation (7, 9). Specifically, this method encourages intrinsic motivation, which develops an internal drive to engage in activities based on an individual's motives, goals, values, and personal interests (12), especially when accompanied by teacher feedback (13).

Concerning the different dimensions engagement involves, it seems that social presence, described as the online student's sense of being and belonging in a course, maybe one of the reasons behind the success of BL (14). In addition, teaching presence also seems to be of great importance since it allows for bridging the transactional distance between student and teacher (15).

Finally, the design of the program instructions comprises those dimensions related to interaction in online environments that can help analyse the educational processes generated in institutions that use this educational modality (16).

BL is an effective method within the health professions (1, 10). Research in this field demonstrates its success in facilitating knowledge acquisition and honing clinical skills across a diverse spectrum of learners and disciplines, particularly in health-related professions, including nursing, medical education, and physician training (1, 10). Furthermore, BL garners preference among students due to its accessibility and adaptability, making it a favoured choice for many (10). However, while BL has made significant strides in these professions, there remains a noticeable gap in its utilization among future health professionals, particularly physiotherapists. On the other hand, the adoption of BL finds a particularly relevant application in healthcare, where the effective management of cardiac diseases and rehabilitation is paramount to patient well-being.

The spectrum of cardiac diseases is vast, and to effectively fulfil their responsibilities, physiotherapists must receive specialized and appropriate training for a comprehensive understanding of cardiac pathology. This knowledge equips them to make informed decisions, tailor rehabilitation programs to individual needs, and support patients recovering from various cardiac conditions. The knowledge, skills, and practices acquired through blended learning can serve as a bridge, addressing the existing educational gap and equipping physiotherapists with the expertise they require in this field of healthcare (17).

In this context, our study aims to assess the effectiveness of a meticulously designed blended learning program tailored to address cardiac pathology. Our focus includes evaluating knowledge acquisition, motivation, engagement, program instructions, and learning behaviours among undergraduate physiotherapy students. Our ultimate goal is to contribute to the comprehensive training of future physiotherapists, subsequently enhancing the quality of cardiac rehabilitation for patients across a broad spectrum of cardiac diseases.

## Material and methods

### Participants and settings

124 undergraduate physiotherapy students (University of Valencia, Spain) were recruited from January 2021 to February 2021. Criteria for participant inclusion were to be studying second year of the Physiotherapy Degree at the aforementioned University and to be willing to participate. Participants with previous heart disease training were excluded to prevent the bias of prior knowledge, as they may have preexisting expectations or biases that could influence their perception or response. From a total of 141 individuals who fulfilled the inclusion criteria, 124 took part in the study. The main reason for exclusion was unwillingness to participate (*n* = 17). The study was carried out at the authors' institution.

### Study design

A prospective study was performed between March 2021 and September 2021. We have considered external factors such as exam and evaluation periods in the study design. Thus, we have selected an intervention period in which the students' teaching load and dedication are reduced. All procedures were conducted according to the Declaration of Helsinki. Participants were informed of the purpose of the study and procedures and provided written informed consent before entering the study. The Institutional Ethics Committee on Human Research approved the study protocol (registered number: IE1623549). The principles of voluntariness, confidentiality and anonymity were respected during the research process. No incentives were provided for participation. The study was registered in Clinicaltrials.gov (NCT05645159) on 25/11/2022.

### Outcome measures

Participants provided demographic information, including age, gender, occupational status, years of previous computer experience, years of previous internet experience and years of previous online learning experience.

We excluded participants with prior training in heart disease to maintain uniformity in our study and ensure that the results accurately reflect the impact of the intervention on people with no prior expertise. Assessments were conducted by a teacher with more than 10 years of experience. The outcomes were as follows:

• *Knowledge acquisition of cardiovascular risk factors and cardiovascular disease,* measured with two questionnaires: (i) “Coronary Artery Disease Education Questionnaire” (CADEQ) (18); and (ii) “Effect of the Dader Method in Cardiovascular Risk of Patients with Risk Factors or Cardiovascular Disease Questionnaire” (EMDADER) (19). CADEQ includes 20 closed-ended questions about medical conditions, risk factors, exercise, nutrition, and psychosocial wellbeing with a single-word answer (yes, no). Total score ranges from 0 to 20 (0 = poorest knowledge, 20 = best knowledge). Cronbach's alpha for the factor subscales was above the acceptable threshold of 0.70 (18). EMDADER is composed of 10 multiple-choice questions with four answer options on coronary artery disease and risk factors, and two questions to report weight and height. Total score ranges from 0 to 10 (0 = poorest knowledge, 10 = best knowledge). The reliability of this instrument has been shown to be between good and excellent (intraclass correlation coefficient of 0.62 to 0.80) (19). Students were not previously informed about the exams to avoid any preparation for the tests.
• *Motivation*, using the Academic Motivation Scale and Attributional Styles Questionnaire (20). The tool is composed of 24 items, using a 7-point Likert scale: from 1 (totally disagree) to 7 (totally agree). The scale integrates three domains related to learning motivation: Intrinsic Motivation (9 items), Performance Motivation (7 items) and Extrinsic Motivation (7 items). The maximum score is 7. The scale has a Cronbach's alpha of 0.70. With regard to the various domains, intrinsic motivation has a Cronbach's alpha of 0.68, performance motivation 0.69 and extrinsic motivation 0.64 (20).
• *Engagement* using the Student Engagement Questionnaire (21)*.* It is a 35-item questionnaire, scored on a five-point Likert scale (1 = totally disagree, 5 = totally agree). The questionnaire is divided into five dimensions: Intellectual Capabilities (items 1–10), Working Together (items 11–16), Teaching (items 17–25), Teacher-student Relationship (items 26–29), and Student-student Relationship (items 30–35). The maximum score of the questionnaire is 5. Cronbach's alpha ranges from 0.75 to 0.89 (21).
• *Design of the program instructions,* using the Community of Inquiry Survey (16)*.* A 34-item questionnaire, scored on a five-point Likert scale (1 = strongly disagree, 5 = strongly agree) that includes three dimensions: (i) Cognitive Presence (items 1–13), related to the degree to which participants are able to construct meaning and knowledge through continuous communication, reflection, and discussion; (ii) Social Presence (items 14–22), related to the ability of participants to identify with the community, communicating and developing interpersonal relationships; (iii) Teaching Presence (items 23–34), referring to the design, guidance and direction, on the part of teachers, of cognitive and social processes with the purpose of achieving the result of meaningful learning in students. The maximum score of the questionnaire is 5. Questionnaire validation shows satisfactory results (Cronbach's alpha being 0.90 for each dimension) (16).
• *Learning behaviours,* including number of downloads, topics visited and total score of questionnaires were retrieved and collected from the institution's Moodle platform Virtual Classroom^TM^, through class progress (22). Students were asked to complete seven multiple-choice online questionnaires, one questionnaire per each theme, the following day after each theoretical class and after having reviewed the online resources related to each theme.

Knowledge was assessed at baseline (T1), at week 4 (T2), at the end of the intervention [week 8 (T3)], and at two follow-up time points (follow-up at 12 weeks (T4) and follow-up at 20 weeks (T5)). Motivation and engagement were assessed at T2 and at T3, whilst design of the program instructions and learning behaviours were measured at the end of the program (T3).

### Intervention

A health allied teacher with over 10 years’ experience in teaching heart disease performed the 8-week BL program. The BL program combined asynchronous online learning modules and scheduled online activities with face-to-face lectures offered at set points during the semester. Participants had autonomy and flexibility for accessing online course content, except for the seven scheduled face-to-face classes. Supplementary Appendix S1 depicts the online resources of the BL program for each theme.

The online resources used were as follows: (i) A *Moodle* platform was utilized as a virtual communication platform, including breaking news, access to the teaching guide, or as a means to solve doubts through discussion forums; (ii) An online syllabus about heart disease composed of seven themes developed from the knowledge considered as basic for health allied students, including topics such as anatomy and physiology of the heart, arrhythmias, hypertension, coronary artery disease, heart failure, shock, endocarditis, myocarditis and pericarditis; (iii) Online videos of international and national scientific societies were used, which aimed at improving the knowledge regarding heart disease, and reinforcing the concepts of heart disease, arrhythmias, nerve conduction, hypertension, angina pain pattern, left ventricular ejection fraction, fluid accumulation in heart failure, cardiogenic shock, etc., In addition, one-minute bullet-point videos were developed by the research team; (iv) Links to websites of international and national scientific societies were used, such as the American Heart Association, the European Society of Cardiology or the Spanish Heart Foundation/World Heart Federation, aimed at reinforcing knowledge acquisition and the concepts of hypertension, coronary artery disease, heart failure, shock, or guidelines for the diagnosis of pericarditis; (v) Podcasts of international and national scientific societies such as the Spanish Cardiac Society or the Spanish Heart Foundation were presented; vi) Online multiple-choice questionnaires were created and personalised comments about the activities were reported by the teachers to give feedback to the students; vii) Mobile apps were used (i.e., *My Heart Anatomy,* and *Ariadna*) to improve knowledge on heart anatomy, or to prevent cardiovascular risk and locate nearby defibrillators; viii) Forums, emails and online tutoring were delivered, by means of an online communication platform (*Blackboard Collaborate*), which is a simple and reliable virtual classroom solution to power online teaching needs. It is a browser-based web conferencing with easy access and high-definition audio and video that enables the students to participate remotely. The tool has been shown to be an efficient means in healthcare and academic environments (1, 10).

### Statistical analysis

Statistical analyses were performed using SPSS v. 24 (IBM SPSS, Inc., Chicago, IL, USA). Descriptive statistics were performed. Continuous variables are shown as mean (standard deviation, SD), and categorical variables as absolute frequency (percentage). Additionally, the inferential analyses of the data were conducted using a one-factor mixed multivariate analysis of variance (MANOVA) having a within-subject factor “time measurements” with five categories (T1, T2, T3, T4 and T5) for knowledge variables. Post-hoc analyses were requested using the Bonferroni correction. Homoscedasticity and sphericity were evaluated using Levene's test and Mauchly's test, respectively. A paired t test was used to investigate motivation changes between T2 and T3, while a Chi-squared test was used to analyse engagement differences between T2 and T3. Furthermore, the correlations between knowledge, motivation, engagement, and design of the program instructions were statistically evaluated using Pearson's correlation coefficient. The *α* level was set at <0.05 for all tests. For the effect size of the continuous variables, Cohen's d was computed, whereby the effect size was rated as follows: small (0.20–0.50), medium (0.50–0.80), or large (>0.80).

## Results

**Figure 1:**
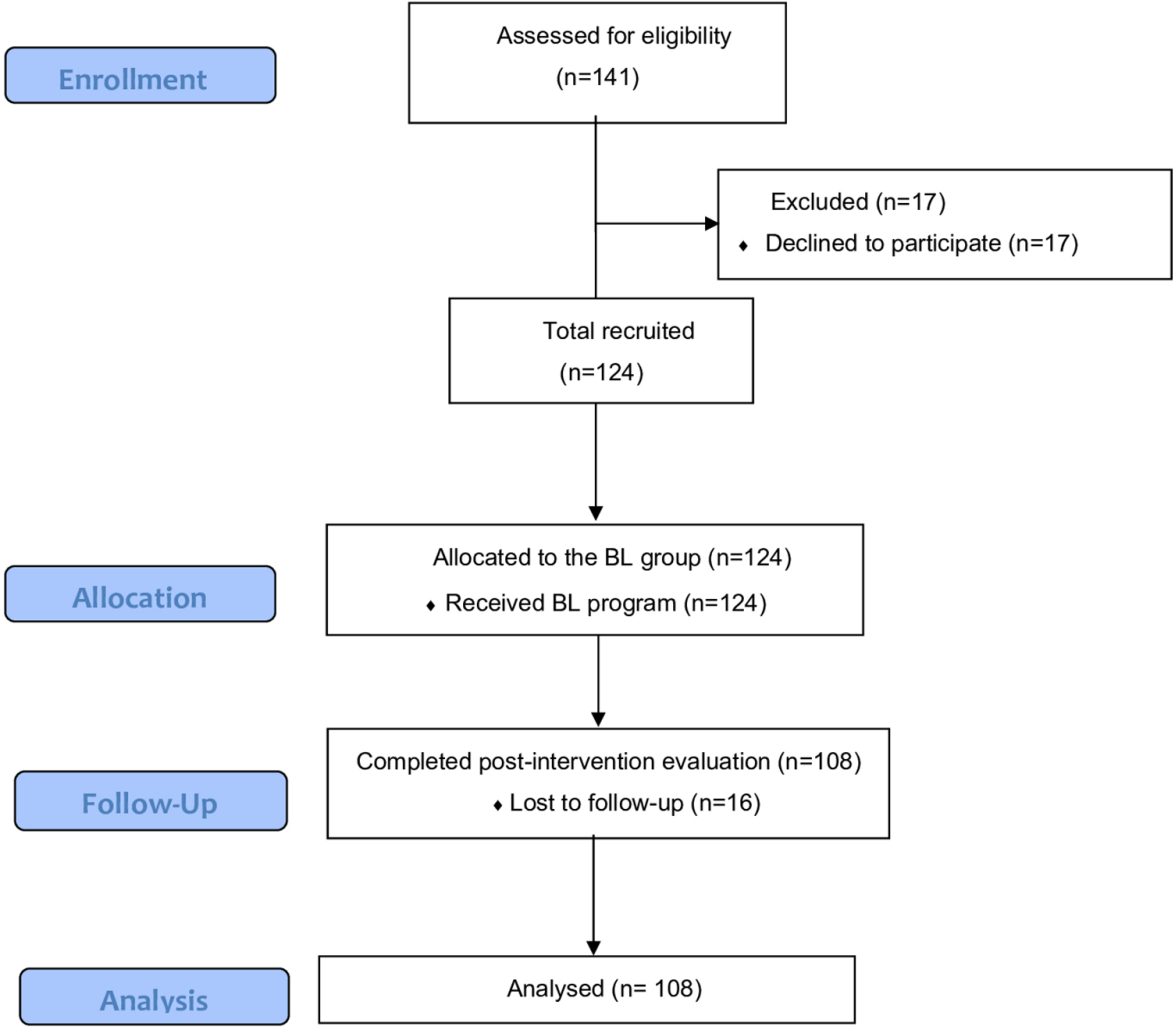
Flow-chart of the study.

The study included 124 participants, of whom 108 (87.10%) completed the study (Figure 1). The mean age was 21.20 ± 4.67) years, and 58.87% were female. All participants presented an online learning experience of 4.28 (3.72) years. Table 1 shows the sociodemographic characteristics of the sample.

**Table 1:** Participants' demographics data.

	*n* = 124
Age (a)	21.20 (4.67)
Sex (b)	
Male	51 (41.13)
Female	73 (58.87)
Employment (b)	
Unemployed	87 (70.16)
Part-time	34 (27.42)
Full-time	3 (2.42)
Previous computer experience (years) (a)	9.82 (4.56)
Previous internet experience (years) (a)	9.90 (3.52)
Previous online learning experience (years) (a)	4.28 (3.72)

Data shown as: (a) mean (standard deviation) and (b) absolute frequency (percentage).

### Knowledge of cardiovascular risk factors and cardiovascular disease

Regarding CADEQ scores (Table 2), there was a significant interaction between intervention and measurement times [*F*(4, 304) = 11.63, *p* < 0.001, *η*^2^ = 42.92]. A gradual and significant increase of knowledge with respect to T1 was observed in pairwise comparisons in the T2 assessments (mean difference = −0.87 ± 0.26), *p* = 0.02, Cohen's *d* = −0.19), in T3 (mean difference = −1.296 ± 0.29), *p* < 0.001, Cohen's *d* = −0.54), in T4 (mean difference = −1.659 ± 0.25), *p* < 0.001, Cohen's *d* = −0.66) and T5 (mean difference = −1.13 ± 0.285), *p* = 0.001, Cohen's *d* = −0.48). Furthermore, a higher score was observed after one-month follow-up than mid-program (mean difference = −0.78 ± 0.23), *p* = 0.01, Cohen's *d* = −0.41).

**Table 2:** Results for knowledge, motivation dimensions, engagement dimensions and comparisons between assessments.

	T1	T2	T3	T4	T5
Knowledge					
CADEQ	13.86 (1.98)	14.73 (1.59)*	15.14 (1.65)*	15.51 (1.26)*^,^**	14.99 (1.56)*
EMDADER	6.91 (1.18)	7.80 (1.20)*	7.48 (0.98)*	8.01 (1.15)*^,^***	7.96 (0.91)*^,^***
Motivation					
Intrinsic Motivation		5.65 (0.69)	5.60 (0.80)		
Extrinsic Motivation		3.85 (0.95)	3.96 (0.95)		
Performance Motivation		4.13 (0.94)	4.24 (0.97)		
Engagement					
Intellectual Capabilities		4.12 (0.53)	4.10 (0.68)		
Working Together		3.92 (0.65)	3.95 (0.66)		
Teaching		4.24 (0.62)	4.21 (0.68)		
Teacher-Student Relationship		4.01 (0.72)	4.00 (0.68)		
Overall Engagement		3.53 (0.74)	3.63 (0.80)		

CADEQ, Coronary Artery Disease Education Questionnaire; EMDADER, Effect of the Dader Method in Cardiovascular Risk of Patients with Risk Factors or Cardiovascular Disease Questionnaire; T1, week 0; T2, week 4; T3, week 8; T4, week 12; T5, week 20.

Data shown as mean (standard deviation).

* *p* < 0.05 vs. T1.

** *p* < 0.05 vs. T2.

*** *p* < 0.05 vs. T3.

Regarding the EMDADER scores, there was a significant interaction between the intervention and the measurement times [*F* (4, 324) = 13.88, *p* < 0.001, *η*^2^ = 55.51]. In addition, the knowledge gained increased significantly with respect to T1 in all subsequent assessments: T2 (mean difference = −0.89 ± 0.19), *p* < 0.001, Cohen's *d* = −0.75), T3 (mean difference = −0.56 ± 0.16), *p* = 0.01, Cohen's *d* = −0.53), T4 (mean difference = −1.10 ± 0.20), *p* < 0.001, Cohen's *d* = −0.94) and T5 (mean difference = −1.05 ± 0.17), *p* < 0.001, Cohen's *d* = −0.90). Significant differences were further observed between T3 and T4 measurements (mean difference = −0.54 ± 0.17), *p* = 0.02, Cohen's *d* = −0.50) and T5 (mean difference = −0.49 ± 0.14), *p* = 0.01, Cohen's *d* = −0.51) (Table 2).

### Motivation

At the end of the program, the Intrinsic Motivation dimension had the best scores (5.60 out of 7 points), followed by the Extrinsic Motivation (4.24 out of 7 points), and, lastly, Performance Motivation (3.96 out of 7 points). As shown in Table 2, there were similar scores at the T2 and T3 assessments (*p* > 0.05).

### Engagement

After the intervention, 87.04% agreed or completely agreed with the items of the Teaching dimension; 81.48% with the items of the Intellectual Capabilities dimension; 78.70% with the Teacher-Student Relationship dimension; 75.92% with the Working Together dimension; and 59.26% with the Student-Student Relationship dimension. Overall Engagement was high (3.98 ± 0.52) out of 5). Scores at the end of the program were similar to those obtained mid-program (*p* > 0.05) (Table 2).

### Design of the blended learning program instructions

Regarding Teaching Presence, more than 95% of the responses indicated "neutral" and above with a mean score of 4.15 ± 0.62. In relation to Social Presence, more than 81.38% of the responses indicated "neutral" and above with a mean score of 3.56 ± 0.8. Finally, with regard to Cognitive Presence, more than 89.66% of the responses indicated "neutral" and above with a mean score of 3.77 ± 0.79 (Data shown in Supplementary Appendix S2).

### Learning behaviours

Table 3 shows the results of the learning behaviours of the sample, including number of downloads, topics visited and total score of online questionnaires, divided by theme and for the whole sample.

**Table 3:** Results for learning behaviours of the sample.

	Theme 1	Theme 2	Theme 3	Theme 4	Theme 5	Theme 6	Theme 7	Total
Number of downloads	11.08 (5.72)	10.99 (6.15)	10.87 (5.66)	9.42 (5.61)	9.75 (5.75)	9.09 (5.25)	9.3 (6.22)	70.51 (36.08)
Topics visited	4.44 (2.13)	4.16 (2.33)	4.64 (2.85)	3.94 (2.33)	4.36 (2.71)	3.25 (2.14)	4.18 (2.78)	28.97 (16.09)
Total score of online questionnaires	7.64 (1.95)	8.15 (2.43)	8.15 (2.42)	7.10 (2.38)	7.56 (2.74)	8.10 (2.01)	7.34 (2.50)	7.67 (1.60)

Data shown as mean (standard deviation).

### Correlations between knowledge, motivation, engagement, and design of the program instructions

We observed a significant positive relationship between Intrinsic Motivation and the following variables: Knowledge [Pearson's Correlation (*r*) = 0.18; *p* = 0.04], Performance Motivation (*r* = 0.31; *p* < 0.001), Engagement (*r* = 0.64; *p* < 0.001), Cognitive Presence (*r* = 0.41, *p* < 0.001), Social Presence (*r* = 0.37; *p* < 0.001) and Teaching Presence (*r* = 0.39; *p* < 0.001); as well as a significant negative relationship with Extrinsic Motivation (*r* = −0.24; *p* = 0.01). In addition, there was a strong correlation between the Engagement and Design of the instructions domains: Cognitive Presence (*r* = 0.71), Social Presence (*r* = 0.65) and Teaching Presence (*r* = 0.70) (*p* < 0.001, in all three comparisons). There was also a low-moderate correlation between Performance Motivation and Social Presence (*r* = 0.21; *p* = 0.01) and Teaching Presence (*r* = 0.19; *p* = 0.03), as well as between Knowledge and Cognitive Presence (*r* = 0.17; *p* = 0.04) (Table 4).

**Table 4:** Results of Pearson's correlation test for all the variables measured at the end of the blended learning programme.

	Knowledge-CADEQ	Knowledge-EMDADER	Intrinsic Mot	Performance Mot	Extrinsic Mot	Engagement	Cognitive Pres	Social Pres	Teaching Pres
Knowledge-CADEQ	1.00								
Knowledge-EMDADER	−0.08	1.00							
Intrinsic Mot	0.18*	0.01	1.00						
Performance Mot	0.11	0.01	0.31***	1.00					
Extrinsic Mot	0.03	−0.09	−0.24**	0.07	1.00				
Engagement	0.06	0.16	0.64***	0.21**	−0.02	1.00			
Cognitive Pres	0.09	0.17*	0.41***	0.00	0.00	0.71***	1.00		
Social Pres	0.04	0.13	0.37***	0.21**	0.14	0.65***	0.62***	1.00	
Teaching Pres	−0.05	0.11	0.39***	0.19*	0.06	0.70***	0.68***	0.65***	1.00

Data shown as Pearson's correlation coefficients. CADEQ, Coronary Artery Disease Education Questionnaire; EMDADER, Effect of the Dader Method in the Cardiovascular Risk of Patients with Risk Factors or Cardiovascular Disease Questionnaire; Intrinsic Mot, Intrinsic Motivation; Performance Mot, Performance Motivation; Extrinsic Mot, Extrinsic Motivation; Cognitive Pres, Cognitive Presence, Social Pres, Social Presence; Teaching Pres, Teaching Presence.

* *p* < 0.05.

** *p* < 0.01.

*** *p* < 0.001.

## Discussion

To the best of our knowledge, this is the first study that evaluates the effect of a heart disease BL program on knowledge, motivation, engagement, design of the program instructions and learning behaviours in future physiotherapists. The proposed BL program is effective in acquiring knowledge about risk factors and cardiovascular disease in the short term (at mid-intervention and at the end of the intervention) and medium term (at 12 and 20 weeks after the start of the intervention). In addition, participants showed a high motivation, engagement, evaluation of the design of the program instructions and learning behaviours. Interestingly, it was observed that the greater the intrinsic motivation, the greater the performance motivation, knowledge, engagement and evaluation of the design of the program instructions.

Regarding the acquisition of knowledge on cardiovascular risk factors and cardiovascular disease, the BL program is effective in the assimilation of theoretical concepts regarding heart disease. These results are consistent with previous studies which concluded that BL is an appropriate learning methodology, equally (4, 23–25) or even more effective than traditional face-to-face learning (6, 9, 26–28). This can be explained by the fact that BL allows integrating the advantages of synchronous learning (face-to-face), for example, obtaining real-time feedback from the teacher and other students, and those of asynchronous learning (e-learning), for example, reviewing the electronic material as many times as necessary and without time restrictions, thus reinforcing learning (1). Our results are consistent with those obtained in another study with health allied students in which there was also unlimited access to an online platform with videos and images (23). In this case, the BL group performed better in its musculoskeletal palpation skills and ultrasound assessment than the traditional learning group, which was only provided with materials such as books and papers. In short, this suggests that BL programs may be a valid tool in the acquisition of knowledge in future physiotherapists.

It has further been described that BL has a high impact on motivation (29). In fact, intrinsic motivation (for example, the item “I enjoy studying because I always discover something new”), had a high score in the present study. This is consistent with the results of the study by McCutcheon et al. (30) in which the BL group obtained higher scores in motivation, attitude and satisfaction than the e-learning group in future nursing professionals. In addition, Lozano-Lozano et al. (25) also suggested that BL provides greater motivation than traditional learning in college Health Science students in a two-week intervention. In contrast, the study by Balakrishnan et al. reported that (26) both groups (i.e., traditional and BL) showed similar levels of motivation. This controversy could be explained by the type of intervention applied, since it seems that more interactive platforms favour motivation, as described in the review by Ødegaard et al. (31). Thus, the online part of the BL group in the study by Balakrishnan et al. (26) only consisted of audio-visual presentations, while our study included a greater number of online resources, such as Moodle, online syllabus, videos, podcasts, apps, online questionnaires, or online tutoring.

In terms of engagement, most of our participants stated that BL is a method that stimulates intellectual skills (i.e., critical thinking, creative thinking, self-managed learning, adaptability and problem solving). A possible reason is that, in BL, the participant acquires an active role and seems to be more involved in the learning process due to the autonomous monitoring of online resources, which in turn allows in-depth review of the subject, enabling participants to interact with the teacher and their peers more effectively (26). This is in line with the findings of Berga et al. (4), who suggested that BL offers pedagogical benefits in terms of increasing participants' confidence in the application of key concepts. In addition, in our study, future professionals stated that BL is a good method in terms of active learning, for syllabus comprehension, evaluation and consistency. Overall, other studies have reported high satisfaction with this methodology referring to the clarity of the instructions, the clarity in the use of learning methods, sufficient time to perform the proposed exercises and improvement of content learning capacity (24, 25).

It is interesting to assess the design of program instructions as it guides teachers towards creating a useful/meaningful learning experience (32). Our findings are consistent with those obtained in the study by Siah et al. (33) addressing a BL program in future nursing professionals. Teaching presence was the most valued dimension, followed by cognitive presence and social presence. In other words, students rated the dimension that refers to design, guidance and direction by the faculty with high scores for achieving significant learning outcomes (32). Therefore, although the time needed by the teachers to prepare the program when using BL is a disadvantage (29), participantś ratings are positive, which should encourage institutions to continue with this approach.

### Limitations and strengths

This study presents a number of limitations that should be taken into account for future research. First, the sample consisted of future physiotherapists from only one institution, which prevents extrapolating the results to other institutions and other health allied professionals. Second, it would have been interesting to compare the results of the BL program against a control group following a traditional learning method, or with an e-learning program. Third, the possibility of assessing motivation, engagement and design of the instructions at the beginning of the intervention and after a follow-up period of could be considered.

Despite the limitations, the present study has several strengths that should be highlighted. On the one hand, studies on BL in higher education are scarce despite its importance based on current technological progress. In fact, to date, this is the first study that addresses a heart disease BL program in future physiotherapists and evaluates the knowledge not only after the intervention, but also mid-program and at two follow-up times in order to determine if the knowledge persists over time. On the other hand, it is highly interesting to evaluate the motivation and engagement, as well as the design of the program instructions, since that in turn favours knowledge acquisition. We further highlight the fact that the BL program carried out has a large amount of online material that is rarely included or scarcely detailed in previous studies.

### Conclusions

The BL program was effective for acquiring knowledge about risk factors and cardiovascular disease. In addition, participants presented high levels of motivation, engagement, evaluation of the design of the program instructions and learning behaviours. Therefore, BL seems to be an effective method for future physiotherapists and may be considered a teaching-learning strategy of choice to be implemented in health allied professions.

## Data Availability

The original contributions presented in the study are included in the article/**Supplementary Material**, further inquiries can be directed to the corresponding authors.

## References

[1] Liu Q Peng W Zhang F Hu R Li Y Yan W. The effectiveness of blended learning in health professions: systematic review and meta-analysis. J Med Internet Res. (2016) 18:e2. 10.2196/jmir.4807 26729058 PMC4717286

[2] Ford PJ Foxlee N Green W. Developing information literacy with first year oral health students. Eur J Dent Educ. (2009) 13:46–51. 10.1111/j.1600-0579.2008.00536.x 19196293

[3] Rao CS. Blended learning: a new hybrid teaching methodology. J Res Scholars Prof Engl Lang Teach. (2019) 13:1–7.

[4] Berga K-A Vadnais E Nelson J Johnston S Buro K Hu R Blended learning versus face-to-face learning in an undergraduate nursing health assessment course: a quasi-experimental study. Nurse Educ Today. (2021) 96:104622. 10.1016/j.nedt.2020.104622 33125980

[5] Owston R York D Murtha S. Student perceptions and achievement in a university blended learning strategic initiative. Internet High Educ. (2013) 18:38–46. 10.1016/j.iheduc.2012.12.003

[6] Kavadella A Tsiklakis K Vougiouklakis G Lionarakis A. Evaluation of a blended learning course for teaching oral radiology to undergraduate dental students. Eur J Dent Educ. (2012) 16:e88–95. 10.1111/j.1600-0579.2011.00680.x 22251359

[7] Li C He J Yuan C Chen B Sun Z. The effects of blended learning on knowledge, skills, and satisfaction in nursing students: a meta-analysis. Nurse Educ Today. (2019) 82:51–7. 10.1016/j.nedt.2019.08.004 31437783

[8] Terry VR Terry PC Moloney C Bowtell L. Face-to-face instruction combined with online resources improves retention of clinical skills among undergraduate nursing students. Nurse Educ Today. (2018) 61:15–9. 10.1016/j.nedt.2017.10.014 29153453

[9] Aguilar-Rodríguez M Marques-Sule E Serra-Añó P Espí-López GV Dueñas-Moscardó L Pérez-Alenda S. A blended-learning programme regarding professional ethics in physiotherapy students. Nurs Ethics. (2019) 26:1410–23. 10.1177/0969733017748479 29458314

[10] Coyne E Rands H Frommolt V Kain V Plugge M Mitchell M. Investigation of blended learning video resources to teach health students clinical skills: an integrative review. Nurse Educ Today. (2018) 63:101–7. 10.1016/j.nedt.2018.01.021 29425738

[11] Rowe M Frantz J Bozalek V. The role of blended learning in the clinical education of healthcare students: a systematic review. Med Teach. (2012) 34:e216–21. 10.3109/0142159X.2012.642831 22455712

[12] Deci EL Ryan RM. Self-determination theory: a macrotheory of human motivation, development, and health. Can Psychol. (2008) 49:182–5. 10.1037/a0012801

[13] Ballouk R Mansour V Dalziel B McDonald J Hegazi I. Medical students’ self-regulation of learning in a blended learning environment: a systematic scoping review. Med Educ Online. (2022) 27:2029336. 10.1080/10872981.2022.2029336 35086439 PMC8803058

[14] Picciano AG. Beyond student perceptions: issues of interaction, presence, and performance in an online course. J Asynchronous Learning Network. (2002) 6:21–40. 10.24059/olj.v6i1.1870

[15] Arbaugh JB Hwang A. Does “teaching presence” exist in online MBA courses? Internet High Educ. (2006) 9:9–21. 10.1016/j.iheduc.2005.12.001

[16] Velázquez BB Gil-Jaurena I Encina JM. Validation of the spanish version of the “community of inquiry” survey. Revista de Educación a Distancia. (2019) 19:4. 10.6018/red/59/04

[17] McMahon SR Ades PA Thompson PD. The role of cardiac rehabilitation in patients with heart disease. Trends Cardiovasc Med. (2017) 27:420–5. 10.1016/j.tcm.2017.02.005 28318815 PMC5643011

[18] Ghisi GdM Grace SL Anchique Cv Gordillo X Fernandez R Quesada D Translation and evaluation of a comprehensive educational program for cardiac rehabilitation patients in Latin America: a multi-national, longitudinal study. Patient Educ Couns. (2021) 104:1140–8. 10.1016/j.pec.2020.10.008 33097358 PMC7550271

[19] Amariles P Pino-Marín D Sabater-Hernández D García-Jiménez E Roig-Sánchez I Faus MJ. Fiabilidad y validez externa de un cuestionario de conocimiento sobre riesgo y enfermedad cardiovascular en pacientes que acuden a farmacias comunitarias de españa. Aten Primaria. (2016) 48:586–95. 10.1016/j.aprim.2016.01.005 27142591 PMC6875968

[20] Lozano AB Rioboo AP Santorum Paz R Carlos Brenlla J Morán Fraga H Barca Enríquez E. LA Escala ceap48: un instrumento de evaluacion de la motivacion academica y atribuciones causales para el alumnado de enseñanza secundaria y universitaria de galicia. n.d.

[21] Gargallo B Suárez-Rodríguez JM Almerich G Verde I Cebrià i Iranzo MÀ. Validación del cuestionario SEQ en población universitaria española. Capacidades del alumno y entorno de enseñanza/aprendizaje. Anales de Psicología. (2018) 34:519–30. 10.6018/analesps.34.3.299041

[22] Juan S. Promoting engagement of nursing students in online learning: use of the student-generated question in a nursing leadership course. Nurse Educ Today. (2021) 97:104710. 10.1016/j.nedt.2020.104710 33341063

[23] Arroyo-Morales M Cantarero-Villanueva I Fernández-Lao C Guirao-Piñeyro M Castro-Martín E Díaz-Rodríguez L. A blended learning approach to palpation and ultrasound imaging skills through supplementation of traditional classroom teaching with an e-learning package. Man Ther. (2012) 17:474–8. 10.1016/j.math.2012.04.002 22579034

[24] Jeganathan S Fleming PS. Blended learning as an adjunct to tutor-led seminars in undergraduate orthodontics: a randomised controlled trial. Br Dent J. (2020) 228:371–5. 10.1038/s41415-020-1332-1 32170259

[25] Lozano-Lozano M Fernández-Lao C Cantarero-Villanueva I Noguerol I Álvarez-Salvago F Cruz-Fernández M A blended learning system to improve motivation, mood state, and satisfaction in undergraduate students: randomized controlled trial. J Med Internet Res. (2020) 22:e17101. 10.2196/17101 32441655 PMC7275253

[26] Balakrishnan A Nair S Kunhikatta V Rashid M Unnikrishnan MK Jagannatha PS Effectiveness of blended learning in pharmacy education: an experimental study using clinical research modules. PLoS One. (2021) 16:e0256814. 10.1371/journal.pone.0256814 34469484 PMC8409684

[27] Moon H Hyun HS. Nursing students’ knowledge, attitude, self-efficacy in blended learning of cardiopulmonary resuscitation: a randomized controlled trial. BMC Med Educ. (2019) 19:48–58. 10.1186/s12909-019-1848-8 31706315 PMC6842519

[28] Shimizu I Nakazawa H Sato Y Wolfhagen IHAP Könings KD. Does blended problem-based learning make Asian medical students active learners?: a prospective comparative study. BMC Med Educ. (2019) 19:147. 10.1186/s12909-019-1575-1 31092243 PMC6521359

[29] Ashraf MA Yang M Zhang Y Denden M Tlili A Liu J A systematic review of systematic reviews on blended learning: trends, gaps and future directions. Psychol Res Behav Manag. (2021) 14:1525–41. 10.2147/PRBM.S331741 34629910 PMC8493276

[30] McCutcheon K O’Halloran P Lohan M. Online learning versus blended learning of clinical supervisee skills with pre-registration nursing students: a randomised controlled trial. Int J Nurs Stud. (2018) 82:30–9. 10.1016/j.ijnurstu.2018.02.005 29574394

[31] Ødegaard NB Myrhaug HT Dahl-Michelsen T Røe Y. Digital learning designs in physiotherapy education: a systematic review and meta-analysis. BMC Med Educ. (2021) 21:48. 10.1186/s12909-020-02483-w 33441140 PMC7805166

[32] Garrison DR Anderson T Archer W. The first decade of the community of inquiry framework: a retrospective. Internet High Educ. (2010) 13:5–9. 10.1016/j.iheduc.2009.10.003

[33] Siah C Lim F Lau S Tam W. The use of the community of inquiry survey in blended learning pedagogy for a clinical skill-based module. J Clin Nurs. (2021) 30:454–65. 10.1111/jocn.15556 33174239

