# Supplementary material for: Effectiveness of a heart disease blended learning program in physiotherapy students: a prospective study
Source: Front Cardiovasc Med. 2023 Nov 22;10:1303997. doi: 10.3389/fcvm.2023.1303997 (PMC10702954; doi:10.3389/fcvm.2023.1303997)
Supplement: Supplementary file 1 [file Table2.docx]

**Appendix**

Appendix 1. Online resources used in each theme

| THEMES | ONLINE RESOURCES |
| --- | --- |
| Theme 1: Introduction to heart disease. Anatomy and physiology of the heart. | Online syllabus  One-minute bullet-point video  Video *How does a normal heart function?* (European Society of Cardiology)  Podcast *Heart-healthy habits* (Spanish Heart Foundation)  Mobile apps *My heart anatomy*  Moodle (*Virtual Classroom*)  Forum  Online multiple-choice questionnaire  Emails  Online tutoring (*Blackboard Collaborate*) |
| Theme 2: Arrhythmias. | Online syllabus  One-minute bullet-point video  Video *Types of arrhythmias* (Spanish Cardiac Society, Spanish Heart Foundation)  Video *Nerve conduction* (European Society of Cardiology)  Mobile apps *Ariadna* (Spanish Cardiac Society)  Moodle (*Virtual Classroom*)  Forum  Online multiple-choice questionnaire  Emails  Online tutoring (*Blackboard Collaborate*) |
| Theme 3: Hypertension. | Online syllabus  One-minute bullet-point video  Video *How to know if you are hypertensive* (Spanish Cardiac Society, Spanish Heart Foundation)  Links to scientific website: *Hypertension* (Spanish Cardiac Society, Spanish Heart Foundation)  Podcast *Hypertension* (Spanish Cardiac Society, Spanish Heart Foundation)  Moodle (*Virtual Classroom*)  Forum  Online multiple-choice questionnaire  Emails  Online tutoring (*Blackboard Collaborate*) |
| Theme 4: Coronary artery disease. | Online syllabus  One-minute bullet-point video  Video *Angina pain pattern* (European Society of Cardiology)  Links to scientific website: *Coronary artery disease* (Spanish Cardiac Society, Spanish Heart Foundation)  Moodle (*Virtual Classroom*)  Forum  Online multiple-choice questionnaire  Emails  Online tutoring (*Blackboard Collaborate*) |
| Theme 5: Heart failure. | Online syllabus  One-minute bullet-point video  Video *Ventricular ejection fraction* (American Heart Association)  Video *How does heart failure cause fluid build-up?* (Heart Failure Association, European Society of Cardiology)  Links to scientific website *Heart Failure* (Spanish Cardiac Society, Spanish Heart Foundation)  Moodle (*Virtual Classroom*)  Forum  Online multiple-choice questionnaire  Emails  Online tutoring (*Blackboard Collaborate*) |
| Theme 6: Shock. | Online syllabus  One-minute bullet-point video  Link to scientific website *Shock code*  Video *Cardiogenic shock* (Spanish Cardiac Society)  Moodle (*Virtual Classroom*)  Forum  Online multiple-choice questionnaire  Emails  Online tutoring (*Blackboard Collaborate*) |
| Theme 7: Endocarditis, myocardial and pericarditis. | Online syllabus  One-minute bullet-point video  Podcast *Myocarditis and pericarditis* (Spanish Cardiac Society, Spanish Heart Foundation)  Link to scientific website *What is infective endocarditis?* (Spanish Cardiac Society, Spanish Heart Foundation)  Link to scientific website *Guidelines for the diagnosis of pericarditis*  Moodle (*Virtual Classroom*)  Forum  Online multiple-choice questionnaire  Emails  Online tutoring (*Blackboard Collaborate*) |

Appendix 2. Results of the design of the blended learning program instructions.

|  | **Strongly disagree**  **n (%)** | **Disagree**  **n (%)** | **Neutral**  **n (%)** | **Agree**  **n (%)** | **Strongly agree**  **n (%)** | **Mean (SD)** |
| --- | --- | --- | --- | --- | --- | --- |
| **Teaching Presence** | | | | | | |
| 1. The instructor clearly communicated important course topics | 2 (1.85) | 1 (0.93) | 8 (7.41) | 46 (42.59) | 51 (47.22) | 4.32 (0.81) |
| 2. The instructor clearly communicated important course goals | 2 (1.85) | 1 (0.93) | 15 (13.89) | 39 (36.11) | 51 (47.22) | 4.26 (0.87) |
| 3. The instructor provided clear instructions on how to participate in course learning activities | 2 (1.85) | 2 (1.85) | 15 (13.89) | 45 (41.67) | 44 (40.74) | 4.18 (0.87) |
| 4. The instructor clearly communicated important due dates/time frames for learning activities | 4 (3.70) | 3 (2.78) | 13 (12.04) | 38 (35.19) | 50 (46.30) | 4.18 (1.00) |
| 5. The instructor was helpful in identifying areas of agreement and disagreement on course topics that helped me to learn | 2 (1.85) | 3 (2.78) | 16 (14.81) | 49 (45.37) | 38 (35.19) | 4.09 (0.88) |
| 6. The instructor was helpful in guiding the class towards understanding course topics in a way that helped me clarify my thinking | 2 (1.85) | 3 (2.78) | 11 (10.19) | 50 (46.30) | 42 (38.89) | 4.18 (0.86) |
| 7. The instructor helped to keep course participants engaged and participating in productive dialogue | 2 (1.85) | 5 (4.63) | 17 (15.74) | 44 (40.74) | 40 (37.04) | 4.06 (0.94) |
| 8. The instructor helped keep the course participants on task in a way that helped me to learn | 2 (1.85) | 3 (2.78) | 20 (18.52) | 45 (41.67) | 38 (35.19) | 4.06 (0.91) |
| 9. The instructor encouraged course participants to explore new concepts in this course | 2 (1.85) | 2 (1.85) | 21 (19.44) | 44 (40.74) | 39 (36.11) | 4.07 (0.89) |
| 10. Instructor actions reinforced the development of a sense of community among course participants | 4 (3.70) | 12 (11.11) | 29 (26.85) | 33 (30.56) | 30 (27.78) | 3.68 (1.11) |
| 11. The instructor helped to focus discussion on relevant issues in a way that helped me to learn | 3 (2.78) | 6 (5.56) | 21 (19.44) | 45 (41.67) | 33 (30.56) | 3.92 (0.99) |
| 12. The instructor provided feedback that helped me understand my strengths and weaknesses relative to the course's goals and objectives | 3 (2.78) | 3 (2.78) | 26 (24.07) | 39 (36.11) | 37 (34.26) | 3.96 (0.98) |
| 13. The instructor provided feedback in a timely fashion | 1 (0.93) | 1 (0.93) | 14 (12.96) | 43 (39.81) | 49 (45.37) | 4.28 (0.80) |
| **Overall Score for Teaching Presence** | _ | _ | _ | _ | _ | 4.15 (0.62) |
| **Social Presence** | | | | | | |
| 14. Getting to know other course participants gave me a sense of belonging in the course | 1 (0.93) | 8 (7.41) | 18 (16.67) | 43 (39.81) | 38 (35.19) | 4.01 (0.95) |
| 15. I was able to form distinct impressions of some course participants | 3 (2.78) | 9 (8.33) | 20 (18.52) | 33 (30.56) | 43 (39.81) | 3.96 (1.08) |
| 16. Online or web-based communication is an excellent medium for social interaction | 23 (21.30) | 23 (21.30) | 15 (13.89) | 30 (27.78) | 17 (15.74) | 2.95 (1.41) |
| 17. I felt comfortable conversing through the online medium | 15 (13.89) | 24 (22.22) | 28 (25.93) | 21 (19.44) | 20 (18.52) | 3.06 (1.31) |
| 18. I felt comfortable participating in the course discussions | 7 (6.48) | 9 (8.33) | 41 (37.96) | 31 (28.70) | 20 (18.52) | 3.44 (1.09) |
| 19. I felt comfortable interacting with other course participants | 4 (3.70) | 6 (5.56) | 24 (22.22) | 44 (40.74) | 30 (27.78) | 3.83 (1.02) |
| 20. I felt comfortable disagreeing with other course participants while still maintaining a sense of trust | 3 (2.78) | 5 (4.63) | 37 (34.26) | 38 (35.19) | 25 (23.15) | 3.71 (0.97) |
| 21. I felt that my point of view was acknowledged by other course participants | 2 (1.85) | 8 (7.41) | 18 (16.67) | 52 (48.15) | 28 (25.93) | 3.89 (0.94) |
| 22. Online discussions help me to develop a sense of collaboration | 13 (12.04) | 18 (16.67) | 30 (27.78) | 30 (27.78) | 17 (15.74) | 3.19 (1.24) |
| **Overall Score for Social Presence** | _ | _ | _ | _ | _ | 3.56 (0.80) |
| **Cognitive Presence** | | | | | | |
| 23. Problems posed increased my interest in course issues | 7 (6.48) | 6 (5.56) | 37 (34.26) | 34 (31.48) | 24 (22.22) | 3.57 (1.10) |
| 24. Course activities piqued my curiosity | 9 (8.33) | 13 (12.04) | 28 (25.93) | 34 (31.48) | 24 (22.22) | 3.47 (1.20) |
| 25. I felt motivated to explore content-related questions | 5 (4.63) | 15 (13.89) | 26 (24.07) | 32 (29.63) | 30 (27.78) | 3.62 (1.17) |
| 26. I used a variety of information sources to explore problems posed in this course | 5 (4.63) | 9 (8.33) | 23 (21.3) | 43 (39.81) | 28 (25.93) | 3.74 (1.08) |
| 27. Brainstorming and finding me resolve content-related questions | 4 (3.70) | 4 (3.70) | 23 (21.30) | 47 (43.52) | 30 (27.78) | 3.88 (0.98) |
| 28. Online discussions were valuable in helping me appreciate different perspectives | 9 (8.33) | 7 (6.48) | 35 (32.41) | 34 (31.48) | 23 (21.30) | 3.51 (1.15) |
| 29. Combining new information helped me answer questions raised in course activities | 5 (4.63) | 4 (3.70) | 26 (24.07) | 44 (40.74) | 29 (26.85) | 3.81 (1.02) |
| 30. Learning activities helped me construct explanations/ solutions | 5 (4.63) | 7 (6.48) | 30 (27.78) | 34 (31.48) | 32 (29.63) | 3.75 (1.09) |
| 31. Reflection on course content and discussions helped me understand fundamental concepts in this class | 4 (3.70) | 4 (3.70) | 25 (23.15) | 45 (41.67) | 30 (27.78) | 3.86 (0.99) |
| 32. I can describe ways to test and apply the knowledge created in this course | 2 (1.85) | 4 (3.70) | 27 (25) | 50 (46.30) | 25 (23.15) | 3.85 (0.88) |
| 33. I have developed solutions to course problems that can be applied in practice | 2 (1.85) | 3 (2.78) | 33 (30.56) | 47 (43.52) | 23 (21.30) | 3.8 (0.87) |
| 34. I can apply the knowledge created in this course to my work or other non-class related activities | 1 (0.93) | 0 (0) | 25 (23.15) | 51 (47.22) | 31 (28.70) | 4.03 (0.78) |
| **Overall Score for Cognitive Presence** | _ | _ | _ | _ | _ | 3.77 (0.79) |

Data shown as absolute frequency (percentage) for categorical variables and mean (standard deviation) for continues variables.
